# Transcriptional profiling links unique human macrophage phenotypes to the growth of intracellular *Salmonella enterica* serovar Typhi

**DOI:** 10.1038/s41598-024-63588-6

**Published:** 2024-06-04

**Authors:** Ruth Schade, Daniel S. C. Butler, Joy A. McKenna, Blanda Di Luccia, Vida Shokoohi, Meagan Hamblin, Trung H. M. Pham, Denise M. Monack

**Affiliations:** 1 Department of Microbiology and Immunology, Stanford University School of Medicine, Stanford, CA 94305 USA; 2 Stanford Functional Genomics Facility, Stanford University, Stanford, CA USA; 3 Department of Pediatrics, Stanford University School of Medicine, Stanford, CA USA

**Keywords:** Bacterial infection, Pathogens, Gene expression

## Abstract

Macrophages provide a crucial environment for *Salmonella enterica* serovar Typhi (*S.* Typhi) to multiply during typhoid fever, yet our understanding of how human macrophages and *S.* Typhi interact remains limited. In this study, we delve into the dynamics of *S*. Typhi replication within human macrophages and the resulting heterogeneous transcriptomic responses of macrophages during infection. Our study reveals key factors that influence macrophage diversity, uncovering distinct immune and metabolic pathways associated with different stages of *S*. Typhi intracellular replication in macrophages. Of note, we found that macrophages harboring replicating *S*. Typhi are skewed towards an M1 pro-inflammatory state, whereas macrophages containing non-replicating *S*. Typhi exhibit neither a distinct M1 pro-inflammatory nor M2 anti-inflammatory state. Additionally, macrophages with replicating *S*. Typhi were characterized by the increased expression of genes associated with STAT3 phosphorylation and the activation of the STAT3 transcription factor. Our results shed light on transcriptomic pathways involved in the susceptibility of human macrophages to intracellular *S*. Typhi replication, thereby providing crucial insight into host phenotypes that restrict and support *S*. Typhi infection.

## Introduction

*Salmonella enterica* serovar Typhi (*S.* Typhi) is a typhoidal serovar of *Salmonella*. Typhoidal serovars differ from most non-typhoidal serovars in several aspects, including their host-tropism as they strictly infect humans. Additionally, typhoidal serovars cause a systemic disease called typhoid fever, rather than the self-limiting gastroenteritis typical of non-typhoidal serovars^1^. The seriousness of this bacterial pathogen as a global health concern is emphasized by the estimated 11.8 million cases of infection and 128,200 deaths in 2016^2^. Furthermore, *S.* Typhi is expected to pose an even greater threat as prevalence of extensively drug resistant strains continues to increase^3^. For these reasons, detangling the complex interactions between *S.* Typhi and host cells is crucial to facilitating the development of new treatments.

*Salmonella* Typhi can overcome immense stress from host immune responses and has been detected in systemic sites. Based on a murine model of typhoidal disease, it is likely that phagocytes recruited to the initial site of infection harbor the bacteria intracellularly and facilitate dissemination throughout the body^4^. Additionally, phagocytes like macrophages also provide an intracellular niche for *S.* Typhi replication^5^. A fascinating complication impacting this intracellular infection strategy is that macrophages can exhibit substantial heterogeneity based on different environmental signals. To classify and simplify this heterogeneity, the M1/M2 polarization paradigm is frequently used, which describes a spectrum of macrophage phenotypes. Macrophages with an inflammatory inclination are classified as being on the M1 side of the spectrum, whereas macrophages with a proclivity toward tissue remodeling and wound healing are designated as being on the M2 side of the spectrum^6,7^.

The significance of macrophage polarization during intracellular infection with *S. enterica* serovar Typhimurium (*S*. Typhimurium), a nontyphoidal serovar, has been well documented in murine models. In vitro*, S.* Typhimurium predominantly replicates in murine M2-like macrophages due, in part, to the *S.* Typhimurium effector protein SteE/SarA^8–11^. This effector recruits the host kinase GSK3 to phosphorylate STAT3 at the tyrosine 705 residue, leading to increased expression of M2 markers^11^.Those findings were supported in vivo when it was found that splenic macrophages in mice infected with wild-type *S.* Typhimurium had higher surface expression of IL-4Ra (M2 marker) at 4 weeks post-infection relative to those infected with a *steE* mutant. Additionally, higher levels of wild-type *S.* Typhimurium were recovered from the livers and spleens of mice compared to the *steE* mutant^12^. While the impact of macrophage heterogeneity during *S.* Typhimurium infection has been studied, macrophage phenotypes resulting from *S*. Typhi infection remain largely unexplored.

In this study, we identify, characterize, and compare the transcriptomic profiles of specific populations of human macrophages arising during *S.* Typhi infection. We show that macrophages containing replicating *S*. Typhi demonstrated enriched expression of genes involved in glycolysis and STAT3 activation, whereas macrophages harboring non-replicating *S*. Typhi had enrichment in genes associated with anaphase and antibacterial alarmins. Our transcriptional profiling is the first to provide insight into human macrophage responses associated with varying stages of intracellular *S*. Typhi replication; thus, providing a valuable resource to better understand these complex interactions.

## Results

### Human macrophages have a heterogeneous response to *S.* Typhi infection

**Figure 1:**
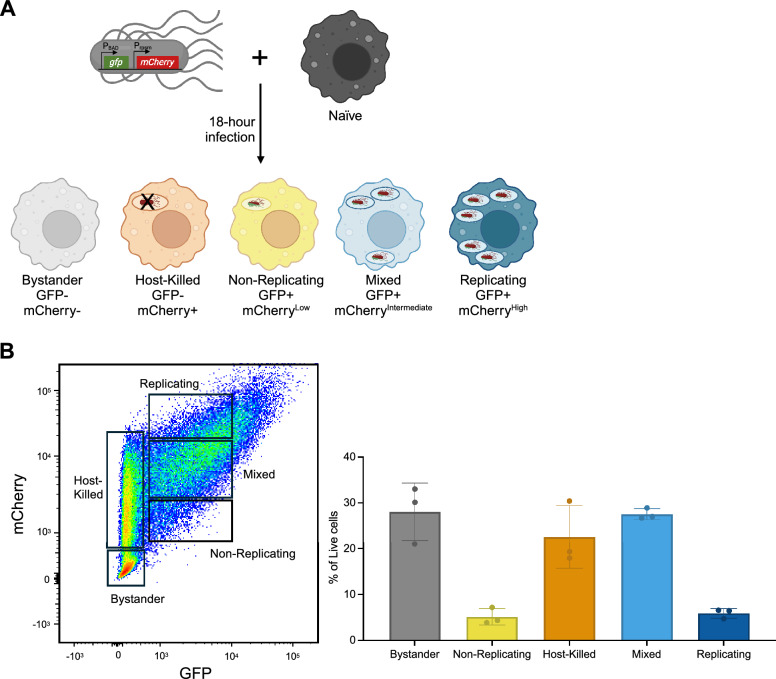
Human macrophages have a heterogeneous response to *S*. Typhi infection. (**A**) Schematic of the experimental design used to assess responses of differentiated THP-1 cells to *S.* Typhi infection 18 h post-infection. Differentiated THP-1 macrophages were infected with *S.* Typhi harboring a fluorescence-dilution reporter plasmid, pFCcGi^14^, with *mCherry* controlled by the constitutively active *rpsM* promoter and *gfp* controlled by the L-arabinose inducible BAD promoter. The GFP and mCherry signals used to classify the THP-1 macrophage populations via flow cytometry are indicated under each macrophage. (**B**) Gating strategy to sort five different populations of differentiated THP-1 macrophages at 18-h post-infection with *S.* Typhi (left panel). The majority of GFP^+^ THP-1 macrophages were mCherry^intermediate^, which were defined as containing a mix of replicating and non-replicating *S*. Typhi. Macrophages with an mCherry signal higher than the mixed population were considered to contain replicating *S*. Typhi, and those with an mCherry signal lower than the mixed population were considered to harbor non-replicating *S*. Typhi. Macrophages with a GFP signal higher than was seen in the mixed population were considered outliers and thus were not collected. Frequency of the five populations of THP-1 macrophages from each of the 3 sorts for RNA-Seq based on mCherry and GFP expression (right panel).

To characterize human macrophage heterogeneity during *S.* Typhi infection, we differentiated THP-1 cells into macrophages via 24 h of exposure to 5 ng/mL PMA followed by 3 days of rest in media without PMA^13^. THP-1 macrophages were infected for 18 h with *S.* Typhi strain Ty2. This particular strain of Ty2 harbored a fluorescence-dilution reporter plasmid (pFCcGi) that indicated the extent of intracellular replication^14^. Briefly, pFCcGi contains a constitutively expressed *mCherry* and an L-arabinose inducible *gfp*. Prior to infection, *S.* Typhi pFCcGi is grown in the presence of L-arabinose whereas during infection L-arabinose is removed so that GFP is ‘diluted’ as bacteria replicate^14,15^. This plasmid also indicates the viability of intracellular *S*. Typhi since GFP is more pH sensitive than mCherry^16^; the lower pH of vacuoles containing dead *S.* Typhi thus degrade GFP, while mCherry is still detectable. Using this system, we defined five populations of THP-1 macrophages challenged with *S.* Typhi: bystander (no intracellular *S*. Typhi), host-killed (all intracellular *S*. Typhi were killed), non-replicating (no replicating intracellular *S*. Typhi), mixed (both replicating and non-replicating intracellular *S*. Typhi), and replicating (replicating intracellular *S*. Typhi) (Fig. 1A). These macrophage populations were sorted by Fluorescence-activated Cell Sorting (FACS) using the gating strategy shown in Fig. 1B.

The challenged macrophages displayed significant diversity in intracellular infection phenotypes, as ~ 30% were bystanders, ~ 5% contained non-replicating *S*. Typhi, ~ 20% contained host-killed *S*. Typhi, ~ 30% contained a mix of replicating and non-replicating *S*. Typhi, and ~ 5% contained replicating *S*. Typhi; the remaining ~ 10% was an outlier population with abnormally high levels of GFP and thus was not collected (Fig. 1B). Naïve macrophages were sorted in tandem as a control. To ensure sufficient transcriptomic coverage, 50,000 cells were collected from each population, and bulk RNA-sequencing yielded an average of ~ 33 million reads per sample. Expression analyses were then completed to identify genes differentially expressed between at least two of the macrophage populations, yielding 10,099 genes for further analysis.

**Figure 2:**
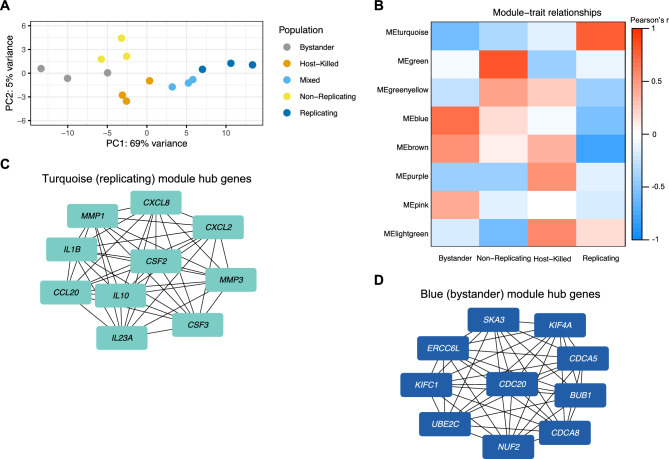
Two modules of co-expressed genes characterize macrophage populations challenged with *S*. Typhi. (**A**) Principal component analysis of THP-1 macrophages challenged with *S.* Typhi. (**B**) Gene modules from Weighted Gene Co-expression Network Analysis that correlated with the different challenged populations and were not impacted by batch effects. Strength and direction of correlations between modules and populations are indicated by the blue to red scale. (**C** and **D**) The top ten hub genes from STRING protein–protein interaction network analysis completed with the most significant genes from the turquoise (replicating) and blue (bystander) modules; significant genes were chosen based on module membership and gene significance scores.

We then tested whether the different states of *S.* Typhi during infection led to transcriptomic heterogeneity in THP-1 macrophages. First, Principal Component Analysis (PCA) was used to visualize the variance in gene expression among naïve and *S*. Typhi-challenged macrophages. In this analysis, PC1 accounted for 92% of the variance in gene expression between the challenged populations and naïve controls, likely due to *S.* Typhi exposure (Supplementary Fig. S2). PC2 demonstrated separation among the challenged populations of macrophages, however, it only accounted for 4% of the variance among all challenged and naïve macrophage populations. Thus, naïve macrophages were excluded from the next PCA analysis to gain more insight into the differences among the challenged populations (Fig. 2A). We found that challenged macrophages clustered based on the different phenotypes of intracellular *S.* Typhi. Macrophage populations harboring no intracellular *S*. Typhi (bystander), non-replicating *S*. Typhi, and host-killed *S*. Typhi were proximal to one another on the left side of the PC1 axis. In contrast, macrophages harboring replicating *S*. Typhi were located on the right side of the PC1 axis (Fig. 2A). This analysis demonstrates that a clonal population of human macrophages can have dramatically different transcriptomic responses to *S.* Typhi infection.

### *S*. Typhi drives gene expression signatures in macrophages

We next investigated which genes comprised the distinct transcriptional macrophage profiles suggested by the PCA. Weighted Gene Co-expression Network Analysis (WGCNA)^17,18^ was used as an unbiased approach to identify gene clusters with highly similar expression patterns (i.e. co-expressed genes) (Supplementary Table S2). Briefly, if a group of genes share a similar expression pattern, they are clustered into a co-expression network or “module”. WGCNA identified 15 co-expression modules in macrophages challenged with *S*. Typhi (Supplementary Fig. S3). Module-trait correlation analysis then identified modules associated with specific macrophage populations with the different populations specified as traits (Supplementary Fig. S3). This analysis revealed high similarity between the macrophage population containing replicating *S*. Typhi and the mixed population containing both replicating and non-replicating *S*. Typhi. Hence, our attention was directed to gene modules that exhibited strong correlations with macrophages containing replicating *S*. Typhi, those with non-replicating *S*. Typhi, or those classified as bystander macrophages (*p* < 0.05, *r* > 0.55) (Fig. 2B, Supplementary Figs. S3, S4).

### The turquoise gene module defines the transcriptomic signature of macrophages harboring replicating *S.* Typhi

We first analyzed the turquoise module (Supplementary Fig. S3), which was strongly associated with macrophages harboring replicating *S*. Typhi (module-trait correlation analysis: *r* = 0.76, *p* = 0.001) (Fig. 2B). To assess the robustness of this module, we calculated the correlation between module membership and gene significance scores for the 4028 genes it contained. This led to an *r* value of 0.7 and *p* < 2.2e^−16^ (Supplementary Fig. S4A). Having established the strength of the turquoise (replicating) module, we pinpointed the most noteworthy subset of genes for a more in-depth analysis. These 376 genes surpassed the threshold value of 0.75 for both module membership and gene significance scores, an approach based on that of Liu et al.^19^.

To identify a transcriptomic signature, we investigated the predicted interactions among the protein products of these 376 genes via STRING protein–protein interaction (PPI) network analysis^20,21^. Gene signatures central to these PPI networks were explored by calculating Maximal Clique Centrality (MCC)^22,23^, which resulted in a set of ten hub genes. MCC selects for hub genes encoding proteins in a network in which all proteins are predicted to interact with each other, thus yielding a collection of hub genes with protein products involved in similar cellular processes. The hub genes identified in the turquoise (replicating) module encoded several cytokines, chemokines, and matrix metalloproteinases (Fig. 2C). Most notable were *IL10*, *IL23A*, *CSF3*, and *CSF2*, which encode cytokines that can regulate activation of STAT3^24^, a transcription factor that can be involved in anti-inflammatory immune responses^10,11^. *CXCL8*, *CXCL2* and *IL1B*, which are chemokines and cytokines involved in neutrophil recruitment/activation^25,26^, were also present. Lastly, these hub genes included the matrix metalloproteinases MMP-1 and MMP-3, which are involved in extracellular matrix (ECM) remodeling^27^. Together, these hub genes reflect a transcriptional signature characteristic of highly activated macrophages^28,29^. Furthermore, the concerted up-regulation of these ten hub genes is unique to macrophages that harbor replicating *S*. Typhi (Supplementary Fig. S5). Thus, these results show that THP-1 macrophages with replicating *S*. Typhi have a distinct transcriptomic signature characterized by cytokine, chemokine, and ECM related genes.

### The blue module contains the transcriptomic signature of the bystander macrophage population

Having identified a distinct transcriptomic signature exclusive to macrophages with replicating *S*. Typhi, we explored modules that could offer transcriptomic signatures specifically linked to the bystander macrophages or the macrophage populations harboring host-killed or non-replicating *S*. Typhi. Although the lightgreen module appeared to exhibit strong correlations with macrophages containing host-killed *S*. Typhi, this module was only comprised of 61 genes. Only one of these genes surpassed the 0.75 threshold set for module membership and gene significance scores (Supplementary Fig. S4B). Therefore, we focused on macrophages harboring non-replicating *S.* Typhi, as well as the bystander macrophage population.

Macrophages containing non-replicating *S.* Typhi were strongly associated with the green module (*r* = 0.8, *p*-value = 3e^−04^) based on module-trait correlation analysis (Supplementary Fig. S3). However, despite having a strong correlation between module membership and gene significance (*r* = 0.64, *p* < 2.2e^−16^), only 26 genes surpassed the thresholds of 0.75 (Supplementary Fig. S4C). Thus, this module was not further explored as a potential gene signature.

The blue and brown modules correlated strongly with the bystander macrophage population (*r* > 0.5, *p*-value < 0.05) (Supplementary Fig. S3). Intriguingly, these modules also demonstrated significant negative correlations with macrophages harboring replicating *S*. Typhi (*r* < − 0.5, *p*-value < 0.05). Further investigation revealed that both modules were robust, with strong relationships between module membership and gene significance (*r* = 0.74 and 0.58 for blue and brown respectively; *p* < 2.2e^−16^ for both modules) (Supplementary Fig. S4D,E). Among the 1954 genes in the blue module, 49 exceeded the threshold score of 0.75 for both module membership and gene significance. In contrast, only 11 of the 1606 genes in the brown module surpassed the thresholds. Thus, we further investigated the blue module to identify a transcriptomic signature for the bystander macrophage population.

To identify predicted functional interactions amongst the 49 genes from the blue (bystander) module, we used STRING protein–protein interaction network analysis^20,21^ (Fig. 2D). All ten hub genes (*CDC20*, *CDCA8*, *CDCA5*, *KIFC1*, *KIF4A*, *SKA3*, *ERCCL6*, *BUB1*, *UBE2C*, *NUF2*) were associated with anaphase processes during mitosis^30–33^, suggesting that these macrophages were actively proceeding through the cell cycle. The expression of all ten hub genes was statistically higher in the bystander population compared to the macrophage populations harboring host-killed and replicating *S.* Typhi. However, this was not observed when comparing the expression of the ten hub genes in bystander macrophages to macrophages harboring non-replicating *S.* Typhi (Supplementary Fig. S6). This suggests that the ten hub genes in the blue (bystander) module represent the transcriptomic signature of the bystander macrophage population; however, this signature may be shared with the macrophages harboring non-replicating *S*. Typhi.

### Macrophages hosting replicating or non-replicating *S.* Typhi exhibit distinct gene expression patterns related to macrophage polarization

We investigated whether biologically significant transcriptomic distinctions might have been overshadowed by less conspicuous phenotypic differences in the macrophage populations harboring host-killed or non-replicating *S*. Typhi, which lacked modules passing the stringent thresholds defined for module membership and gene significance. To do this, we identified the genes differentially expressed between macrophages harboring replicating *S*. Typhi versus bystander macrophages or macrophages with host-killed or non-replicating *S*. Typhi (Supplementary Table S3). Unsurprisingly, a comparison of bystander macrophages and macrophages harboring replicating *S*. Typhi had the most significant differentially expressed genes with 1150. Differential expression analysis comparing macrophages harboring replicating and host-killed *S*. Typhi identified 639 differentially expressed genes, which reflects the presence of killed versus live intracellular *S*. Typhi. Notably, macrophages harboring replicating compared to non-replicating *S*. Typhi had 677 significant differentially expressed genes (significance defined by the adjusted *p*-value < 0.01). We were intrigued by the substantial number of genes differentially expressed between macrophages harboring replicating versus non-replicating *S*. Typhi despite both populations possessing live intracellular *S*. Typhi. This suggested that the intracellular environment of these two macrophage populations differed in respect to permitting or inhibiting *S*. Typhi replication. This finding led us to further explore transcriptomic differences between macrophages harboring replicating or non-replicating *S.* Typhi, with a focus on genes involved in M1 (pro-inflammatory) or M2 (anti-inflammatory/wound healing) polarization.

**Figure 3:**
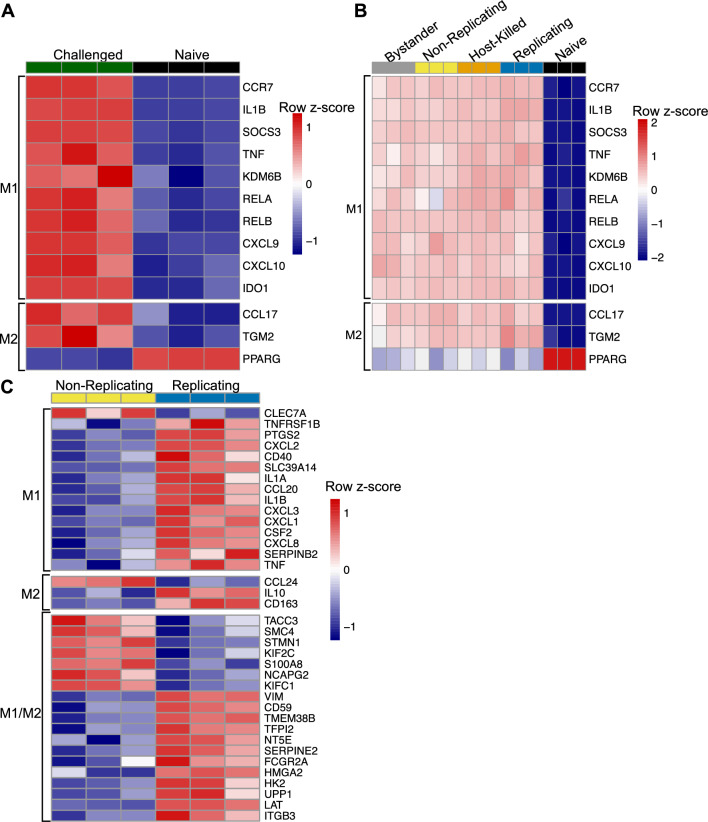
Macrophage polarization phenotypes are associated with differences in intracellular *S*. Typhi infection and replication. (**A**) Heatmap displaying gene expression in naïve hMDMs and hMDMs challenged with *S*. Typhi for 8-h. The hMDM gene expression dataset is from a publicly available RNA-Seq dataset (PRJNA721701)^37^. The genes included in this heatmap are commonly used to define M1 and M2 polarization in hMDMs^38,39^. (**B**) Heatmap showing gene expression in the different populations of THP-1 macrophages challenged with *S*. Typhi and naive THP-1 macrophage controls. The genes included in this heatmap are commonly used to define M1 and M2 polarization in hMDMs ^38,39^. (**C**) Heatmap showing expression of M1, M2, and M1/M2 genes from our compiled list (Supplementary Table S6). Genes shown here were significantly differentially expressed between the replicating and non-replicating populations (adjusted *p*-value < 0.01) For all heatmaps, the fill color is based on relative expression of each gene determined by row z-scores.

Macrophage polarization to an M1 or M2 state prior to infection can skew host responses, thereby impacting permissibility to intracellular bacterial replication^34,35^. To ensure that our THP-1 macrophages were not skewed towards a given polarization state prior to infection, we compared expression levels of M0-associated genes in our naïve THP-1 macrophages versus those challenged with *S*. Typhi. This analysis revealed higher expression of *CD33*, *ITGAM*, *HAVCR2*, *CD36*, *MERTK*, *SIGLEC5*, *SIGLEC9*, and *FCGR1A* in naïve THP-1 macrophages, all of which encode proteins shown by Forrester et al.^36^ to be associated with M0 THP-1 macrophages. Having confirmed an M0 state in our naïve THP-1 macrophages, we determined if the macrophage polarization phenotypes from our THP-1 macrophages challenged with *S*. Typhi were similar to those from other *S*. Typhi macrophage infection studies. We compared the expression of genes in our challenged versus naïve THP-1 macrophages with a publicly available RNA-Seq dataset from human monocyte-derived macrophages (hMDMs) challenged with *S*. Typhi^37^. To facilitate this, we compiled a list of genes exhibiting different transcriptional levels in M1 versus M2 polarized hMDMs based on prior studies^38,39^ (Supplementary Table S4). We assessed expression of these genes in the hMDM dataset and identified those with significant differences in expression between macrophages challenged with *S*. Typhi and naïve macrophages (Supplementary Table S5, Fig. 3A).

We then used these markers to determine if our THP-1 macrophages had similar gene expression patterns (Fig. 3B). Our analysis showed that ten out of the eleven M1 marker genes and two out of the four M2 marker genes in this hMDM marker list were more highly expressed in both hMDMs and THP-1 macrophages challenged with *S*. Typhi. None of the hMDM M1 marker genes exhibited higher expression in naïve hMDMs or THP-1 macrophages. The only M2-associated gene expressed to a higher extent in naïve hMDMs or THP-1 macrophages was *PPARG*.

Having established that classic hMDM markers suggest an M1 polarization phenotype in both hMDMs and THP-1 macrophages during *S*. Typhi infection, we characterized our THP-1 macrophage phenotypes further. Thus, we manually curated a list of M1 and M2 markers (Supplementary Table S6) from previous THP-1 macrophage polarization studies^40–43^. Genes with an increased expression in response to both M1- and M2-polarizing stimuli were termed “M1/M2” markers and reflect a general activation response. This list mirrored the patterns of the hMDM macrophage polarization marker gene expression in our THP-1 macrophages (Supplementary Table S7). M1-associated marker gene expression increased in THP-1 macrophages challenged with *S*. Typhi compared to naïve controls, with no substantial differences in the extent of M2- or M1/M2-associated marker gene expression in these challenged THP-1 macrophages. Given the consistency in polarization phenotypes demonstrated by our THP-1 macrophages using established hMDM and THP-1 macrophage markers, we used our assembled list of THP-1 macrophage polarization markers to compare THP-1 macrophages harboring replicating versus non-replicating *S*. Typhi (Supplementary Table S8, Fig. 3C).

Macrophages harboring replicating *S*. Typhi exhibited enrichment in fourteen M1-, two M2-, and twelve M1/M2-asssociated genes relative to the non-replicating population (Fig. 3C). These 14 M1 genes included *IL1B*, *TNF*, and *CD40*, which are typical markers used to define M1 polarization in hMDMs and THP-1 macrophages^38–43^. Murine macrophages harboring replicating *S*. Typhimurium have been shown to upregulate the murine M2 markers *Il4ra* and *Arg1*^8,9^. *ARG1* transcripts were not detected in our naïve or challenged THP-1 macrophages. While the IL-4 receptor is not a commonly used M2 marker for human macrophages, *IL4R* expression was higher in macrophages harboring replicating, as opposed to non-replicating, *S*. Typhi (log_2_ fold change = 0.53, adjusted *p*-value = 0.02) (Table S3). The enriched expression of M1 marker genes in macrophages harboring replicating *S*. Typhi was intriguing as this contrasts with the pronounced M2-like polarization state documented in murine macrophages harboring replicating *S*. Typhimurium^8,9^. In comparison, macrophages harboring non-replicating *S*. Typhi had higher expression of one M1-, one M2-, and seven M1/M2-associated genes relative to macrophages with replicating *S*. Typhi (Fig. 3C). This suggests that macrophages harboring replicating *S*. Typhi are skewed towards an M1 state, whereas macrophages containing non-replicating *S*. Typhi exhibit a relatively general activation state (M1/M2).

### Identifying the macrophage phenotypes linked to intracellular *S*. Typhi replication

**Figure 4:**
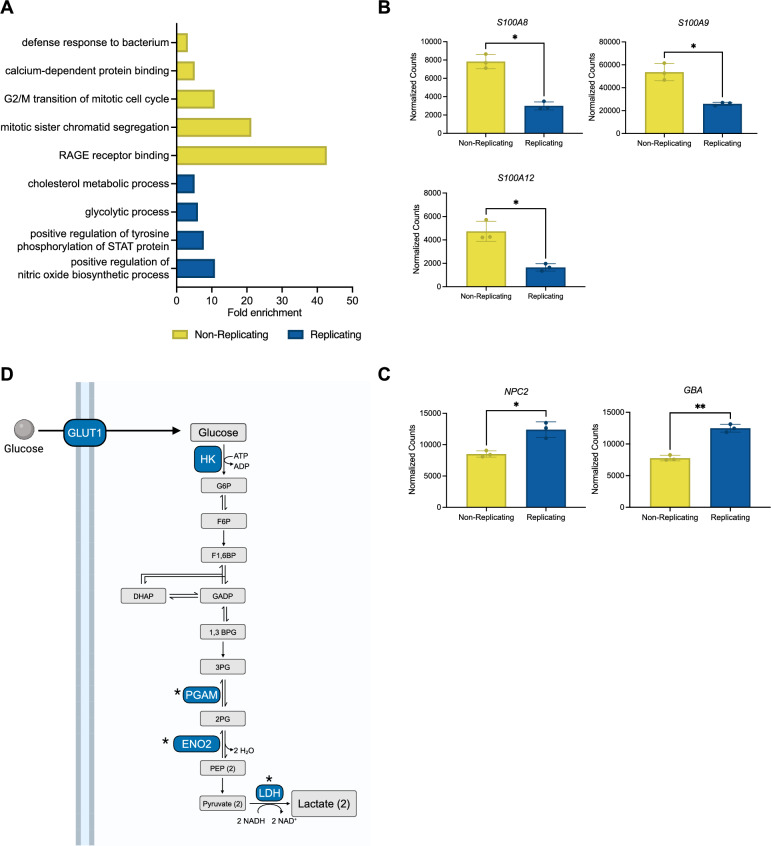
Macrophages harboring replicating versus non-replicating *S*. Typhi show enrichment in different immune and metabolic pathways. (**A**) Significant GO terms from pathway analysis using genes significantly differentially expressed between the replicating and non-replicating populations (adjusted *p*-value < 0.01). Pathway analysis was completed with DAVID^66,67^, which determines fold enrichment by calculating the ratio of genes in the user’s list in relation to the genes in the designated pathway. (**B**) Normalized count data for top genes in RAGE receptor binding and calcium-dependent protein binding pathways that are enriched in macrophages harboring non-replicating bacteria. (**C**) Normalized count data for the top genes in the cholesterol metabolism pathway. (**D**) Schematic representation of glucose uptake and glycolysis pathways. Grey boxes represent metabolites. Proteins encoded by genes that are differentially expressed in macrophages harboring replicating *S*. Typhi are shown in blue boxes. Asterisks indicate genes with significantly different expression between macrophages housing replicating versus non-replicating *S*. Typhi based on paired t-tests (* indicates *p* < 0.05).

Macrophage polarization is shaped by a broad range of cellular processes; thus, we utilized pathway enrichment analysis to identify functional distinctions between macrophages harboring replicating versus non-replicating *S*. Typhi. By utilizing the extensive collection of gene sets provided by the Gene Ontology (GO) database^44,45^, we identified overrepresented pathways within the lists of genes differentially expressed between macrophage populations with replicating versus non-replicating *S*. Typhi. This analysis revealed gene enrichment in multiple pathways linked to crucial host responses to infection (Fig. 4A, Supplementary Table S9).

Macrophages containing non-replicating *S*. Typhi exhibited enrichment in GO term pathways that included: RAGE receptor binding, mitotic sister chromatid segregation, G2/M transition of mitotic cell cycle, calcium-dependent protein binding, and defense response to bacterium (Fold enrichment scores of 42.7, 21.24, 10.84, 5.16, and 3.18 respectively). Interestingly, the RAGE receptor binding, calcium-dependent protein binding, and defense response to bacterium pathways all included *S100A8/9/12*, which encode alarmins that signal through TLR4 and RAGE (Fig. 4B)^46^. Additionally, the G2/M phase transition and sister chromatid segregation pathways were linked to mitotic processes. These findings suggest that the macrophages containing non-replicating *S*. Typhi were characterized by elevated expression of alarmin genes and genes involved in mitosis, particularly anaphase.

The macrophage population containing replicating *S*. Typhi exhibited enrichment in GO term pathways that included: cholesterol metabolic process, positive regulation of tyrosine phosphorylation of STAT protein, positive regulation of nitric oxide synthesis, and glycolytic process (Fold enrichment scores of 5.15, 7.74, 10.9, and 6.07 respectively) (Fig. 4A). The positive regulation of nitric oxide synthesis pathway included the M1 marker genes *IL1B*, *TNF*, and *PTGS2*, thus reflecting the M1 polarization phenotype associated with this population. Of further interest were the cholesterol and glucose metabolic pathways due to the strong interconnection between host cell metabolism and immune processes^47–49^. Among the cholesterol metabolism genes, *NPC2* and *GBA* exhibited the most statistically significant differences in expression between macrophages harboring replicating versus non-replicating *S*. Typhi (Fig. 4C). Both of these genes encode lysosomal proteins involved in lipid trafficking and processing^50^. The glucose metabolism genes included *SLC2A1*, which encodes glucose transporter 1^51^, and genes encoding enzymes involved in glycolysis, such as *HK2* (hexokinase 2), *ENO*2 (enolase-2), and *PGAM1* (phosphoglycerate mutase 1)^52^, along with *LDHA*, which encodes lactate dehydrogenase^52^ (Fig. 4D, Supplementary Fig. S7). This analysis suggests an augmentation in cholesterol and glucose metabolism in the macrophages harboring replicating *S*. Typhi.

### Macrophages harboring replicating *S*. Typhi display heightened STAT3 activation and elevated expression of cytokine genes upstream of STAT3 activation

**Figure 5:**
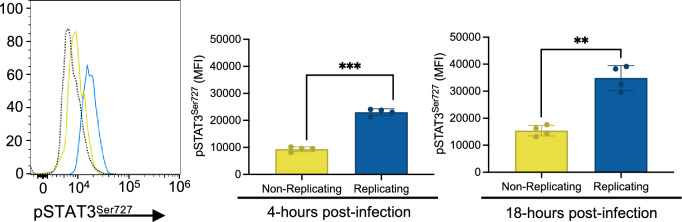
Macrophages containing replicating *S*. Typhi had elevated phosphorylation of STAT3 at the serine 727 residue. THP-1 macrophages were infected with *S*. Typhi pFCcGi. At 4- and 18-h post-infection (hpi), cells were collected and STAT3 phosphorylation at Ser727 was measured via phospho-flow cytometry^54^. The histogram depicts fluorescence of pSTAT3^Ser727^ in macrophages containing non-replicating *S*. Typhi (yellow), macrophages containing replicating *S*. Typhi (blue), and macrophages serving as an unstained control (dotted line). Median fluorescence intensity of pSTAT3^Ser727^ in the two populations of challenged macrophages at 4- and 18-hpi is shown in the bar plots to the right of the histogram. Data shown includes samples from independent experiments; n = 2 for both experiments.

Due to our identification of the positive regulation of tyrosine phosphorylation of STAT protein pathway in macrophages harboring replicating *S.* Typhi, and the importance of STAT3 during *S.* Typhimurium infection, we chose to experimentally characterize this pathway in macrophages infected with *S.* Typhi. STAT3 can be phosphorylated at several residues, and it is known that LPS triggers STAT3^Ser727^ phosphorylation, leading to glycolytic flux associated with classical macrophage activation^53^. Thus, we used phospho-flow cytometry^54^ to assess STAT3 phosphorylation at Ser727 at 4- and 18-hpi (Fig. 5). Our findings revealed that at both time points, macrophages containing replicating *S*. Typhi exhibited higher levels of phosphorylation at Ser727 than macrophages harboring non-replicating *S*. Typhi.

**Figure 6:**
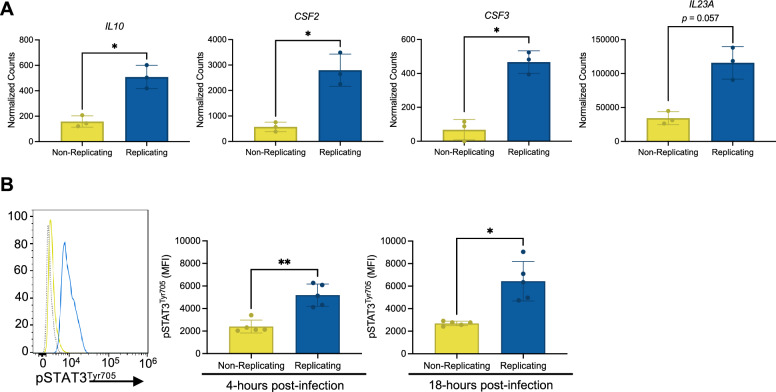
Macrophages containing replicating *S*. Typhi had elevated phosphorylation of STAT3 at the tyrosine 705 residue. (**A**) Normalized count data from RNA-Seq on macrophages containing non-replicating *S*. Typhi versus replicating *S*. Typhi. The genes shown encode cytokines that are upstream regulators of STAT3 phosphorylation at the Tyr705 residue^24^. (**B**) THP-1 macrophages were infected with *S*. Typhi pFCcGi. At 4- and 18-h post-infection (hpi), cells were collected and STAT3 phosphorylation at Tyr705 was measured via phospho-flow cytometry^54^. The histogram depicts fluorescence of pSTAT3^Tyr705^ in macrophages containing non-replicating *S*. Typhi (yellow), macrophages containing replicating *S*. Typhi (blue), and macrophages serving as an unstained control (dotted line). Median fluorescence intensity of pSTAT3^Tyr705^ in the two populations of challenged macrophages at 4- and 18-hpi is shown in the bar plots to the right of the histogram. Data shown includes samples from independent experiments; n = 2 and n = 3.

Both WGCNA and differential gene expression analysis identified a correlation between macrophages harboring replicating *S*. Typhi and cytokines involved in regulating STAT3 tyrosine phosphorylation. *IL10*, *IL23A*, *CSF2*, and *CSF3*, identified as hub genes from the turquoise (replicating) module, are known regulators of STAT3 activation resulting in Tyr705 phosphorylation^24^ (Fig. 6A). *IL23A* and *CSF2* were also included in the list of genes associated with the positive regulation of tyrosine phosphorylation of STAT protein GO term (Fig. 4A). Consequently, we used phospho-flow cytometry to validate STAT3 phosphorylation at Tyr705 (Fig. 6B). Our findings demonstrated that macrophages harboring replicating *S*. Typhi had higher levels of STAT3^Tyr705^ phosphorylation compared to macrophages containing non-replicating *S*. Typhi at 4- and 18-hpi. In summary, these results indicate that *S.* Typhi replicates in macrophages that display increased STAT3 activation.

## Discussion

Despite causing over 100,000 annual deaths^2^, comprehensive studies investigating immune responses to *S.* Typhi at the cellular level are scarce. Most studies related to typhoid fever have used a murine model of *S.* Typhimurium infection to investigate pathogenesis^1^; however, while *S.* Typhimurium causes a systemic disease in mice resembling that caused by *S.* Typhi in humans, these two serovars have sizeable genetic differences. A comparison of *S*. Typhimurium LT2 and *S*. Typhi CT18 found that these common reference strains shared only 89% of genes, with differences in multiple virulence factors^55^. Thus, the *S.* Typhimurium murine infection model is unable to capture every aspect of the mechanisms by which *S.* Typhi interacts with human immune cells. To our knowledge, this study represents the first unbiased attempt to delineate distinctions among human macrophages based on the replication and survival of intracellular *S.* Typhi.

Our data revealed significant heterogeneity in both intracellular bacterial replication and macrophage transcriptomic responses during *S*. Typhi infection. Macrophages harboring replicating *S*. Typhi were characterized by genes encoding cytokines upstream of STAT3 activation, and all ten hub genes from WGCNA were reflective of a highly activated state. In contrast, the bystander population was characterized by higher expression of genes involved in anaphase. This finding is intriguing, particularly considering a recent study showing that *S*. Typhi was better able to infect THP-1 macrophages when carrying a wild-type copy of the typhoid toxin gene, whose expressed product can arrest cells in the G2 cell cycle phase^56^. It is worth noting that macrophages containing non-replicating *S*. Typhi expressed these anaphase genes in a similar manner to bystander macrophages. This observation further implies that variations in cell cycle phases among macrophages may contribute to the permissiveness of a given macrophage to intracellular *S*. Typhi infection.

While the bystander macrophages and the macrophages harboring replicating *S*. Typhi demonstrated the greatest transcriptomic differences, over 600 genes were found to be differentially expressed between macrophages harboring replicating versus non-replicating *S*. Typhi. This finding intrigued us, as both populations harbored live *S*. Typhi and were exposed to the same extracellular stimuli. Consequently, we focused on identifying potential functional differences impacting permissibility to intracellular *S*. Typhi replication. Notably, macrophages hosting replicating *S.* Typhi exhibited higher expression levels of M1-associated genes compared to those with non-replicating bacteria, and neither population displayed substantial expression of M2-associated genes. This observation was surprising, as murine macrophages harboring replicating *S*. Typhimurium have been characterized by higher expression of M2-associated genes^8,9^, and *S*. Typhimurium employs an effector to induce M2 polarization^10–12^. While we did not characterize THP-1 macrophages with a higher-than-average GFP signal from *S.* Typhi pFCcGi, we speculate that this outlier population may be of interest to future studies since these macrophages likely have a pronounced M1 phenotype due to the high burden of intracellular *S.* Typhi.

Pathway analysis revealed several pathways that are potentially pivotal in determining the permissiveness of a macrophage to intracellular *S.* Typhi replication. Enrichment in anaphase-related processes in macrophages harboring non-replicating *S*. Typhi aligned with the hub genes observed in the blue (bystander) module identified by WGCNA, thus underscoring the significant role cell cycle phases may play in influencing permissibility to intracellular *S*. Typhi replication. High expression of *S100A8/9/12* in macrophages with non-replicating *S*. Typhi contributed to enrichment in the RAGE signaling and calcium-dependent protein binding pathways. S1008/9, also called calprotectin, can function as an alarmin, eliciting inflammatory immune responses in neighboring cells through RAGE and TLR signaling^46^. Additionally, In vitro studies have shown that macrophages treated with butyrate exhibited enhanced clearance of intracellular *S.* Typhimurium due to increased *S100A8/9* expression^57^. Our data suggest that calprotectin may similarly contribute to creating a less permissive niche for *S*. Typhi replication within macrophages.

In contrast, macrophages housing replicating *S*. Typhi exhibited increased expression of genes involved in pathways that potentially support the survival and replication of intracellular bacteria. Among the cholesterol metabolism genes highlighted by pathway enrichment analysis, *GBA*, which encodes glucocerebrosidase, emerged as a potential key player in fostering a more hospitable environment within *Salmonella* containing vacuoles (SCVs). Studies utilizing a zebrafish model of *M. marinum* infection demonstrated that *GBA* deficiency led to the accumulation of bactericidal glucosylsphingosine in lysosomes^58^, which impeded growth of *M. marinum*, even at sub-bactericidal concentrations^58^. If macrophages containing non-replicating *S*. Typhi harbor higher concentrations of glucosylsphingosine within SCVs compared to macrophages harboring replicating bacteria, this may contribute to reducing permissiveness to *S.* Typhi replication.

Macrophage glucose metabolism is intricately linked to immune responses; for instance, LPS-induced phosphorylation at the Ser727 residue of pSTAT3 has been observed to mediate increased glycolytic flux^53^. Macrophages harboring replicating *S*. Typhi also exhibit higher levels of STAT3 phosphorylation at Ser727, coupled with increased expression of enzymes integral to glycolysis. This emphasis on glycolysis-related genes holds significance, as aerobic glycolysis is typically recognized as a hallmark of M1 macrophages and a pivotal component of the response to LPS^53^. This connection between M1 polarization and the population with replicating *S*. Typhi is intriguing, given that M1 macrophages are traditionally regarded as highly inflammatory and bactericidal. However, the increased expression of *LDHA*, encoding lactate dehydrogenase, suggests that macrophages harboring replicating *S*. Typhi may have higher intracellular lactate concentrations. While *S.* Typhimurium does not use host lactate as a nutrient source within macrophages, it was recently demonstrated that lactate can modulate *S.* Typhimurium SPI-2 gene expression in murine macrophages^59^. This interplay between lactate and expression of SPI-2 effectors, especially SteE, was found to facilitate intracellular replication^59^. Although this relationship has not been explored in the context of *S.* Typhi infection, the heightened expression of genes linked to glycolysis and lactate production could potentially contribute to similar processes involved in intracellular replication.

SteE serves a crucial role in steering macrophage responses to facilitate intracellular *S.* Typhimurium persistence in murine macrophages^11,12^. It operates by recruiting a host kinase, GSK3, to phosphorylate STAT3 at the Tyr705 residue, thereby promoting a more M2-like state^10,11^. The heightened phosphorylation of STAT3 at the Tyr705 residue in THP-1 macrophages harboring replicating *S*. Typhi is particularly intriguing, considering that *S*. Typhi lacks SteE or homologous proteins. The importance of phosphorylation at this residue is further underscored by the four hub genes in the turquoise (replicating) module that encode regulators of STAT3 activation.

Heterogeneous immune responses to bacterial infection are likely more common than previously considered. Indeed, our data suggest that macrophage permissibility to infection is attributed to cell-to-cell differences despite our macrophages being derived from a clonal line. Of note, we observed a negative correlation between macrophages expressing anaphase-related genes and their permissibility to *S*. Typhi infection and intracellular replication, which suggests that macrophage cell cycles impact *S.* Typhi uptake. Furthermore, our study strongly demonstrates that *S.* Typhi also drives macrophage heterogeneity. In future studies, we are interested in probing if intracellular populations of metabolically active or inactive *S.* Typhi can drive macrophage polarization as previously shown for *S.* Typhimurium in murine macrophages^9^.

At human body temperature, *S.* Typhi up-regulates the production of an important virulence factor known as the Vi capsular polysaccharide^60^, which aids *S.* Typhi evasion of opsonization by complement to avoid complement-mediated phagocytosis^61,62^. In order to increase human macrophage uptake of *S.* Typhi, we cultured and opsonized *S*. Typhi at 22 °C to avoid Vi antigen production^60^ as done previously^15^. This strategy allowed for robust statistical analyses and may provide insights into macrophage interactions with *S*. Typhi clinical isolates that do not produce Vi antigen^63^. Similarly, for technical feasibility, we used an MOI of 150 to ensure that 70% of THP-1 macrophages would phagocytose *S*. Typhi. We recognize that a high MOI may promote a more pro-inflammatory innate immune response in the *Salmonella* exposed THP-1 macrophages than that of a lower MOI. However, the expression of classic M1 and M2 markers in our THP-1 data are consistent with a prior study of naïve versus *S.* Typhi challenged hMDMs that used an MOI of 10^37^. Future studies exploring alternative infection conditions will build on our work and contribute to a more comprehensive model of *S.* Typhi infection in human macrophages.

This study reveals significant heterogeneity in human macrophage responses to *S.* Typhi, intricately tied to the intensity of intracellular replication. As we move forward with mechanistic studies, the roles of the cell cycle and alarmin expression merit further investigation regarding their role in constraining intracellular *S.* Typhi replication. Delving into the metabolic nuances of challenged macrophages, particularly in the realm of lactate production, promises to unveil crucial insights into the permissive niche for *S.* Typhi replication. Lastly, the association of STAT3 activation with intracellular *S*. Typhi replication in macrophages raises intriguing questions, hinting at the possible existence of a novel effector orchestrating STAT3 activation.

## Methods

### Cell culture

THP-1 cells from ATCC (American Type Culture Collection: TIB-202) were routinely cultured in suspension in Roswell Park Memorial Institute (RPMI) 1640 Medium containing l-glutamine and supplemented with 10% heat-inactivated FBS and (2 mM) l-glutamine at 37 °C in 5% CO_2_. Only THP-1 cells below passage 10 were used. For seeding and differentiation, cells were seeded in 15 cm, 9 cm petri dishes, or 6-well plates that had been coated with human plasma-derived fibronectin per the manufacturer’s instructions. For 15 cm dishes, 7 × 10^7^ cells were seeded; for the 9 cm petri dishes, 1 × 10^7^ cells were seeded; for the 6-well plates, 3 × 10^6^ cells were seeded per well. THP-1 cells were differentiated into macrophage-like adherent cells via treatment with phorbol 12-myristiate-12 acetate (PMA) (5 ng/mL) for 24 h at 37 °C in 5% CO_2_. After 24 h of exposure to PMA, the differentiation media was aspirated and replaced with fresh cell culture media without PMA, allowing cells to rest for 72 h prior to further use.

### *S.* Typhi infection of THP-1 macrophages and sample collection

*S*. Typhi strain Ty2^60^ harboring the pFCcGi fluorescence-dilution reporter plasmid^14^ was used for all macrophage infections. pFCcGi was a gift from Sophie Helaine & David Holden (Addgene plasmid # 59,324; http://n2t.net/addgene:59324; RRID:Addgene_59324). The *S*. Typhi harboring pFCcGi was grown at 22 °C in Miller’s Luria Broth (LB) supplemented with aromix (40 µg/mL L-phenylalanine, 40 µg/mL L-tryptophan, 10 µg/mL p-aminobenzoic acid, and 10 µg/mL dihydroxybenzoic acid) and 50 µg/mL carbenicillin and 0.4% L-arabinose. At OD_600_ 0.7, bacteria were collected, spun down (1150 rcf for 3 min), and resuspended to opsonize with room temperature 25% AB human serum in PBS for 30 min. Differentiated THP-1 cells were infected with the opsonized *S*. Typhi Ty2 at an MOI of 150. One-h post-infection (hpi), THP-1 cells were treated with gentamicin (100 µg/mL) for 1 h, followed by gentamicin (20 µg/mL) in RPMI containing l-glutamine and supplemented with 10% heat-inactivated FBS and l-glutamine (2 mM) at 37 °C in 5% CO_2_ for an additional 17 h.

At 18 hpi, the media was aspirated and replaced with enough trypLE Express to cover the THP-1 cells; this was done with both naïve and infected samples. The THP-1 cells were incubated at 37 °C with 5% CO_2_ for 15 min and then harvested with a cell scraper (Sarstedt). Harvested cells were washed twice with FACS buffer (PBS + 2 mM EDTA) at 4 °C. The samples were then resuspended in RPMI + l-glutamine supplemented with 10% FBS and GlutaMax (2 mM), and DAPI was added 12 min prior to Fluorescence-activated Cell Sorting as a viability marker. Naïve and infected THP-1 cells were treated the same way as they were prepared for sorting. Three biological replicates of naïve and infected THP-1 macrophages were prepared in total. At 18 hpi, most of the THP-1 macrophages were alive, with averages of 78% viability in macrophages challenged with *S*. Typhi and 88% viability in naïve macrophages.

### RNA extraction, library prep, and sequencing

To sort cells, we used a FACSymphony S6 sorter with continuous 4 °C cooling and 130 µM nozzle size. Cells were gated and sorted based on viability (DAPI), along with GFP and mCherry signals from pFCcGi (Fig. 1A). The gating strategy for classifying GFP^+^ THP-1 macrophages as containing replicating, mixed, or non-replicating *S*. Typhi was informed by previous studies^8,9^ that used pFCcGi to track *S*. Typhimurium intracellular replication in murine macrophages. In those studies, macrophages harboring *S.* Typhimurium pFCcGI (GFP^+^) had mCherry^+^ signals that ranged from low, intermediate, and high. The mcherry^+^ signals were translated to represent macrophages that harbor non-replicating, mixed, or replicating populations of *S.* Typhimurium pFCcGi. Sorted cells were dispensed directly into Qiazol (Qiagen). A total of 50,000 cells were collected from each of our defined macrophage populations. Samples were then flash frozen with ethanol and dry ice, and bulk-RNA was extracted using chloroform followed by the Qiagen miRNeasy Micro Kit with an on-column DNase treatment. Libraries were made using the KAPA Stranded mRNA-Seq Kit, with KAPA mRNA Capture Beads following the standard protocol with 100 ng of total RNA input. Prepared libraries were then run on an Agilent High Sensitivity bio analyzer-chip to determine the library fragment size distribution and molarity. Equal moles of individual libraries were then pooled for final QC and sequencing on Illumina NovaSeq 6000. RNA extraction, library preparation, and sequencing were completed by the Stanford Genomics Service Center. All fastq files generated by this sequencing can be accessed through NCBI’s Gene Expression Omnibus under the GEO Series accession number GSE267899.

### RNA-Seq quality control and alignment

Fastq reads were processed with the nf-core RNA-seq pipeline (Version 3.9) using the defaults for this pipeline. Sample strandedness was specified as “reverse”. Quality control was done with FastQC (Version 0.11.9). TrimGalore! (Version 0.6.5) was used to filter out adaptors and poor-quality reads. STAR (Version 2.7.10b) mapped the reads to the human genome (GRCh37 from the iGenomes reference), and Salmon (Version 1.9.0) was used to quantify transcripts. Supplementary Table S1 provides the raw count data from this quantification.

### RNA-Seq data processing and DESeq2 analysis

The raw count data from Salmon underwent batch correction using ComBat-Seq^64^ from the sva R package (Version 3.46.0), which uses negative binomial regression specifically developed for RNA-Seq count data. The batch effect specified for correction was the replicate number for each sample, thus accounting for biological differences from sorting on different days. The batch-corrected counts data were then normalized using the DESeq2^65^ (Version 1.38.3) R package^65^, which also assigned Hugo Gene Nomenclature Committee (HGNC) symbols to each gene. Differential expression analyses were done using DESeq2 to identify genes differentially expressed between our five populations of macrophages challenged with *S*. Typhi and naïve controls, with significance defined as an adjusted *p*-value < 0.01. PCA was also completed with DESeq2.

### Weighted gene co-expression network analysis

Weighted gene co-expression network analysis was done using the WGCNA R package^17^ (Version 1.72-1). The genes included in this analysis had significant differential expression in at least one binary comparison between *S*. Typhi-challenged populations, including analyses of each *S*. Typhi-challenged population versus the naïve population. A soft threshold power of eight was chosen since it is the lowest power at which the scale-free topology index reaches 0.9. That power was then used in constructing an adjacency matrix, which was then transformed to produce the topological overlap matrix (TOM). Genes were then clustered into modules according to TOM-based dissimilarity, with a minimum module size of thirty genes (Supplementary Table S2). Module eigengenes were calculated, and modules were clustered hierarchically. Modules were merged based on a dissimilarity threshold of 0.3.

The resulting modules were correlated with traits of interest, which included the different populations, the biological replicate, and whether the population contained live intracellular *S*. Typhi. Modules with a Pearson’s correlation coefficient (*r*) > 0.1 or < − 0.1 in relation to biological replicates were excluded from further analysis to ensure the modules not being impacted by batch effects. Modules of interest were selected based on the relationship between module membership and gene significance scores. Module membership was calculated using the signed eigengene-based connectivity algorithm. In calculating gene significance, the traits being considered were the different macrophage populations. The gene significance scores utilized for selecting modules of interest were those for the population correlating most strongly with the module being evaluated; gene significance values considered for the turquoise module were those relating to the replicating population, whereas gene significance values considered for the blue module were those relating to the bystander population. Genes in the turquoise and blue modules had significant correlations between module membership and gene significance scores, these two modules had the greatest number of genes above the threshold scores of 0.75. This threshold was used to identify which genes to consider in further analyses.

### Protein–protein interaction (PPI) network analysis and hub gene identification

Key genes selected from the turquoise and blue modules based on gene significance and module membership scores were included in PPI network analysis completed with STRINGapp in Cytoscape^23^, with a confidence score threshold of 0.4. Following construction of these networks, the top ten hub genes in each network were identified based on the Maximal Clique Centrality calculation by the cytoHubba plugin^22^ in Cytoscape. Unbiased Euclidean clustering analyses were completed with the pheatmap (Version 1.0.12) R package. Level of expression shown in the heatmap corresponds to row z-scores calculated from log(gene expression + 1).

### Macrophage polarization gene analysis

Markers of M1/M2 hMDM polarization were compiled from studies comparing gene expression between hMDMs stimulates with interferon-gamma and LPS (M1) versus hMDMs stimulated with IL-4 (M2)^38,39^. Markers of THP-1 macrophage polarization were compiled based on qRT-PCR, Luminex, and proteomics data^40–43^. These M1, M2, and M1/M2 marker lists were used to sort the genes differentially expressed between macrophage populations into their respective polarization categories. This analysis was done with publicly available RNA-Seq data (PRJNA721701) from naïve hMDMs and hMDMs challenged with *S*. Typhi for 8-h^37^, as well as with the sorted populations of THP-1 macrophages challenged with *S*. Typhi for 18-h. Heatmaps were constructed with the Pheatmap package as described above, however, Euclidean clustering was not used.

### Binary comparisons of the macrophage populations harboring replicating versus non-replicating *S*. Typhi

DAVID^66,67^ was used to identify GO term pathways^44,45^ enriched for significantly differentially expressed genes between the replicating and non-replicating populations. Enrichment was determined based on the fold enrichment score calculated by DAVID, which is the ratio of the percentage of genes in a pathway that are present in the dataset versus the percentage of genes in a pathway that would be expected as background. Pathways of interest were selected based on significant *p*-values, their ranking in the GO term hierarchy (pathways at the lowest levels, which are the most specific, were prioritized), and if they are biologically relevant to macrophage infection. Redundancy in pathways meeting these criteria was addressed by selecting the pathway containing more differentially expressed genes. This approach yielded five pathways of interest associated with macrophages harboring non-replicating bacteria and four pathways of interest associated with macrophages harboring replicating bacteria. Genes of interest contained in these pathways were selected based on over-representation in multiple pathways, or membership in biologically relevant pathways of interest (i.e., metabolic pathways or pathways known to impact macrophage immune responses). Paired t-tests were done with GraphPad Prism (Version 10.0.3) software to assess significant differences in gene expression between the non-replicating and replicating populations, with *p* < 0.05 considered significant.

### Phospho-flow cytometry

THP-1 cells were grown, seeded, PMA-differentiated, and infected as previously described but without fibronectin coating. Both naïve and infected PMA-differentiated THP-1 cells were harvested by cell scraping at 4- and 18-hpi, and viability staining was done with fixable blue dye (Invitrogen; 1:250 dilution). The cells were incubated at 37 °C in BD Cytofix/Cytoperm™ Fixation/Permeabilization Kit followed by further fixation using methanol (− 20 °C, 15 min). Then, the cells were stained with Brilliant Violet 421™ anti-STAT3 Phospho (Tyr705) (BioLegend; 1:50 dilution) or Brilliant Violet 421™ anti-STAT3 Phospho (Ser727) (BioLegend; 1:50 dilution). Samples were analyzed using the Cytek Aurora spectral flow cytometer, and the data were then further analyzed using FlowJo (Version 10.8.0).

### Statistics and data visualization

Data were analyzed and visualized in GraphPad Prism (Version 10.0.3) software or using the R packages mentioned above. Paired *t*-tests and one-way repeated measures ANOVAs were used to determine significance, with *p* < 0.05 considered significant. BioRender.com was utilized to create the schematic of macrophage populations harboring *S*. Typhi with pFCcGi (Fig. 1A) and the glycolysis schematic (Fig. 4D).

### Supplementary Information

Supplementary Information 1.

Supplementary Table S1.

Supplementary Table S2.

Supplementary Table S3.

Supplementary Table S4.

Supplementary Table S5.

Supplementary Table S6.

Supplementary Table S7.

Supplementary Table S8.

Supplementary Table S9.

## Data Availability

The data discussed in this publication have been deposited in NCBI’s Gene Expression Omnibus^68^ and are accessible through GEO Series accession number GSE267899 (https://www.ncbi.nlm.nih.gov/geo/query/acc.cgi?acc=GSE267899). The raw counts from RNA-Seq transcript quantification by Salmon (Version 1.9.0) are provided in Supplementary Table S1. This counts file indicates the ensembl ID for each transcript, and the columns of the table correspond to the sample the counts are from. All code used in this study are derived from prior publications.
