# Supplementary material for: Transcriptional profiling links unique human macrophage phenotypes to the growth of intracellular Salmonella enterica serovar Typhi
Source: Sci Rep. 2024 Jun 4;14:12811. doi: 10.1038/s41598-024-63588-6 (PMC11150401; doi:10.1038/s41598-024-63588-6)
Supplement: Supplementary file 1 — Supplementary Information 1. [file 41598_2024_63588_MOESM1_ESM.pdf]

Supplementary Information for

Transcriptional profiling links unique human macrophage phenotypes to the growth of intracellular *Salmonella enterica* serovar Typhi

Ruth Schade[1], Daniel S.C. Butler[1†], Joy A. McKenna[1†], Blanda Di Luccia[1], Vida Shokoohi[2], Meagan Hamblin[1], Trung H.M. Pham[3], Denise M. Monack[1*]

1. Department of Microbiology and Immunology, Stanford University School of Medicine, Stanford, CA 94305

2. Stanford Functional Genomics Facility, Stanford University, Stanford, CA, USA

3. Department of Pediatrics, Stanford University School of Medicine, Stanford, CA, USA

*Address correspondence to Denise M. Monack, dmonack@stanford.edu

[†]These authors contributed equally to this work. Author order is alphabetical.

This PDF file includes:

Captions for Supplementary Tables S1-S9

Supplementary Figures S1-S6

Supplementary Tables

Supplementary Table S1: Raw count data from transcript quantification.

Supplementary Table S2: Genes in each module from WGCNA. This table lists the genes assigned to each module from Weighted Gene Co-expression Network Analysis. The ensemble ID and HGNC symbol are provided for each gene.

Supplementary Table S3: Pair-wise differential gene expression analysis. The results of differential gene expression analyses completed with DESeq2 are provided in the different sheets of this file. The specific analyses provided include: binary comparisons of each of the challenged populations versus the naïve controls and binary comparisons of each of the challenged populations versus macrophages harboring replicating *S*. Typhi.

Supplementary Table S4: Classic M1 and M2 hMDM polarization markers. List of commonly used M1 and M2 polarization markers from studies characterizing human monocyte-derived macrophages stimulated with IFNγ + LPS (M1) or IL-4 (M2).

Supplementary Table S5: Expression of significantly differentially expressed M1 and M2 hMDM marker genes in data from Wang *et al*. DESeq2 normalized expression values for the hMDM M1 and M2 marker genes significantly differentially expressed (adjusted *p*-value < 0.01) between naïve hMDMs and hMDMs challenged with *S*. Typhi. The counts data used for this analysis is from Wang *et al*, NCBI BioProject accession PRJNA721701. The raw sequencing data were acquired from SRA and processed in the same way as our THP-1 sequencing data.

Supplementary Table S6: Compiled list of M1 and M2 polarization markers for THP-1 macrophages, as well as markers of a general activation state in THP-1

macrophages. These markers were compiled from several studies that treated THP-1 macrophages with IFNγ + LPS (M1) or IL-4 + IL-13 (M2). Markers that showed increased expression in response to both M1 and M2 polarizing cytokine stimulation are designated “activated”, which corresponds to “M1/M2” in Figure 3.

Supplementary Table S7: Expression of significantly differentially expressed M1, M2, and general activation THP-1 macrophage marker genes in naïve and challenged THP-1 macrophages. DESeq2 normalized expression values for the THP-1 macrophage M1 and M2 marker genes. significantly differentially expressed (adjusted *p*-value < 0.01) between naïve THP-1 macrophages and THP-1 macrophages challenged with *S*. Typhi.

Supplementary Table S8: Differential expression analysis comparing expression of THP-1 macrophage polarization markers in THP-1 macrophages harboring replicating versus non-replicating *S*. Typhi.

Supplementary Table S9: Top GO Term pathways associated with genes differentially expressed between macrophages harboring replicating versus non-replicating *S*. Typhi. The differentially expressed genes contained in the top GO Term pathways are provided, as well as the *p*-values and fold enrichment values from the pathway enrichment analysis completed with DAVID.

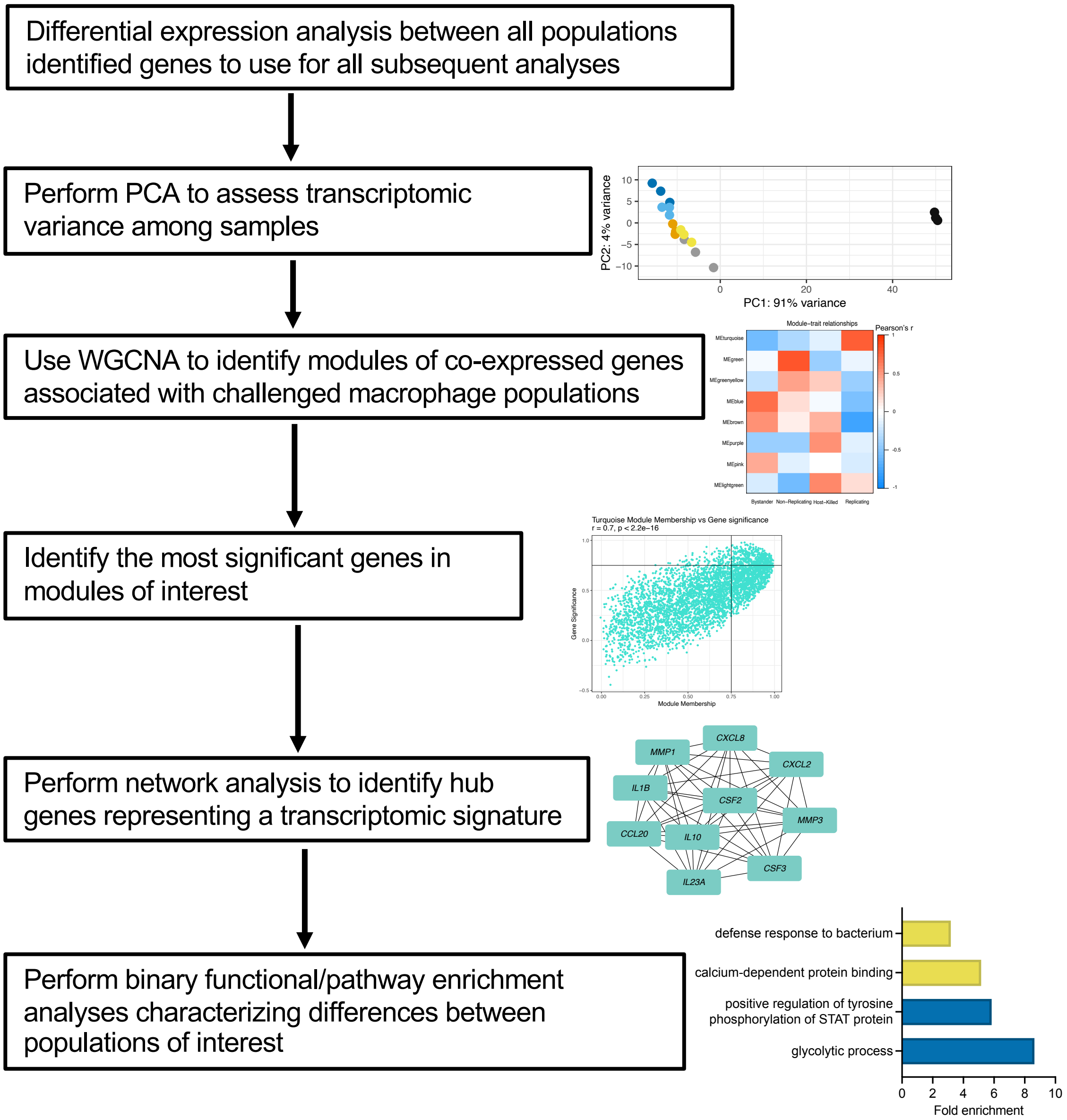

Figure S1: Overview of the approach to transcriptomic analysis.

This flowchart depicts the workflow utilized to analyze transcriptomic data from THP-1 macrophages challenged with *S.* Typhi. First, differential gene expression analyses identified genes significantly differentially expressed between at least two macrophage populations; genes with significant adjusted *p*-values were included in subsequent analyses. Next, Principal component analysis (PCA) assessed transcriptomic variance among macrophages challenged with *S*. Typhi and naïve controls. Weighted gene co-expression network analysis (WGCNA) was performed to identify transcriptomic signatures of different populations of macrophages challenged with *S*. Typhi. Module membership and gene significance scores were calculated for all genes in modules of interest; genes that exceeded score thresholds of 0.75 were selected for further investigation. These genes were used for STRING protein-protein network analysis, and Maximal Clique Centrality was calculated to identify the top ten hub genes. Binary analyses then identified differences in pathway enrichment between macrophages harboring replicating versus non-replicating bacteria.

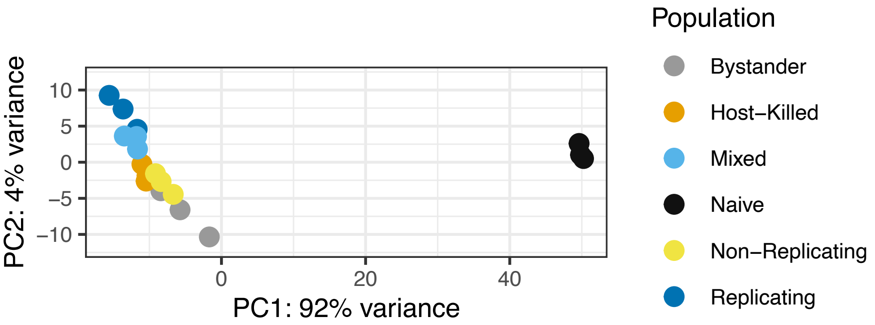

Figure S2: Host transcriptomic patterns correspond to infection status.

Principal component analysis of RNA-Seq performed on viable naïve (unexposed) and *S*. Typhi-challenged THP-1 macrophages collected via Fluorescence-activated Cell Sorting.

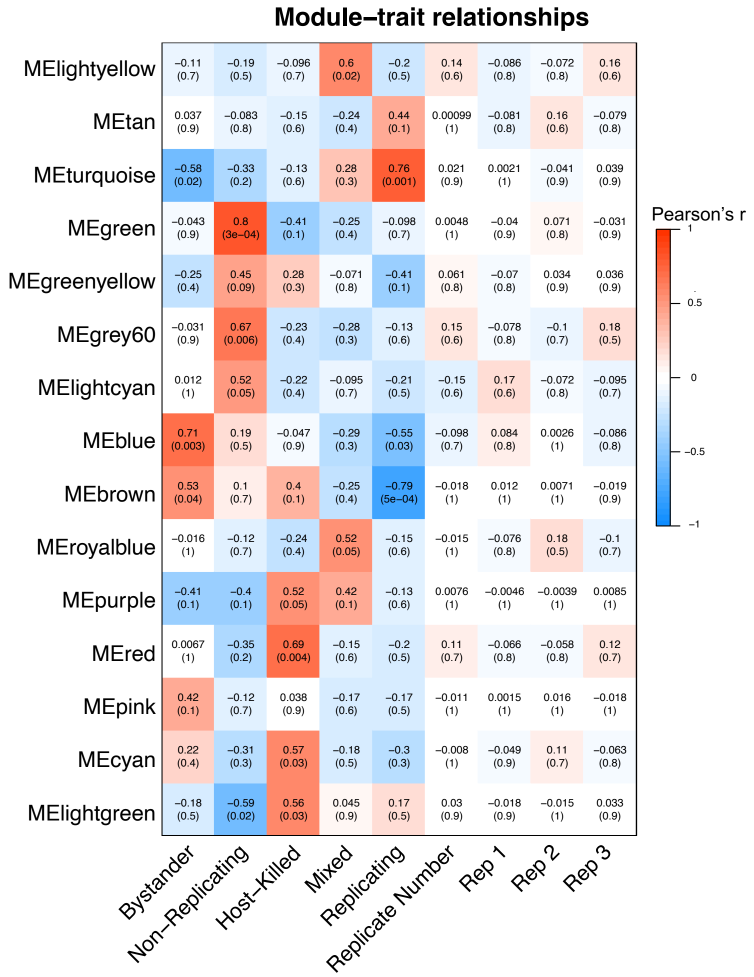

Figure S3: WGCNA identified clusters of co-expressed genes relevant to intracellular infection and replication.

All gene modules from Weighted Gene Co-expression Network Analysis (y-axis) were correlated with various sample traits (x-axis). Individual biological replicates are labeled "Rep 1", "Rep 2", and "Rep 3". "Replicate Number" refers to which replicate each sample was in. Pearson correlation coefficients and *p*-values for each module trait relationship are shown.

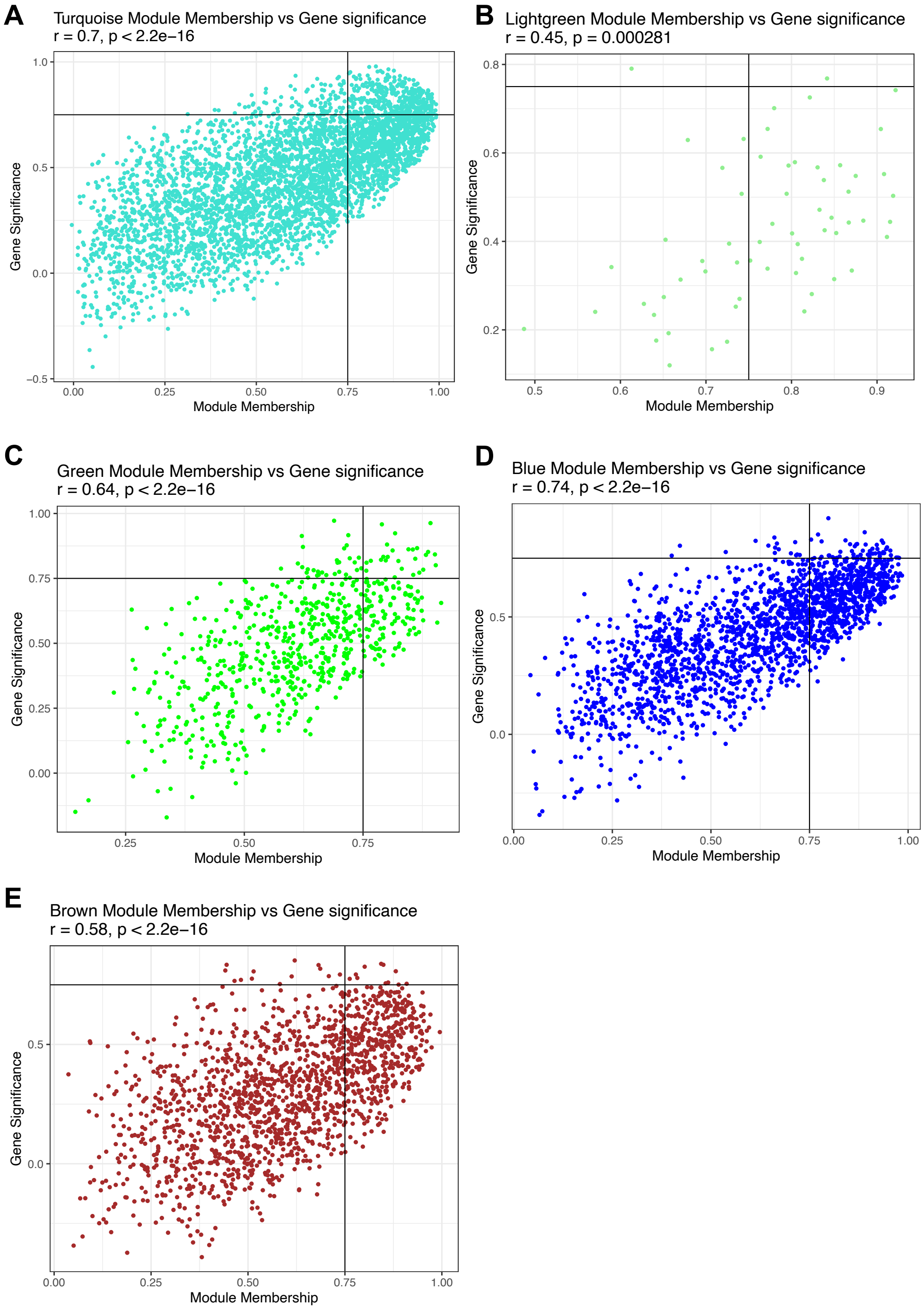

Figure S4: Module membership and gene significance scores indicate the most significant modules associated with specific challenged populations.

Scatter plots with dots representing each gene contained in the A) turquoise, B) lightgreen, C) green, D) blue, and E) brown modules. Module membership scores for genes in each module were calculated using the signed eigengene-based connectivity algorithm in the WGCNA R package. The gene significance scores for each module are based on the significance of each gene with relation to the challenged population most strongly correlated with that module. Key genes for further exploration were identified based on thresholds set at 0.75 for both gene significance and module membership scores.

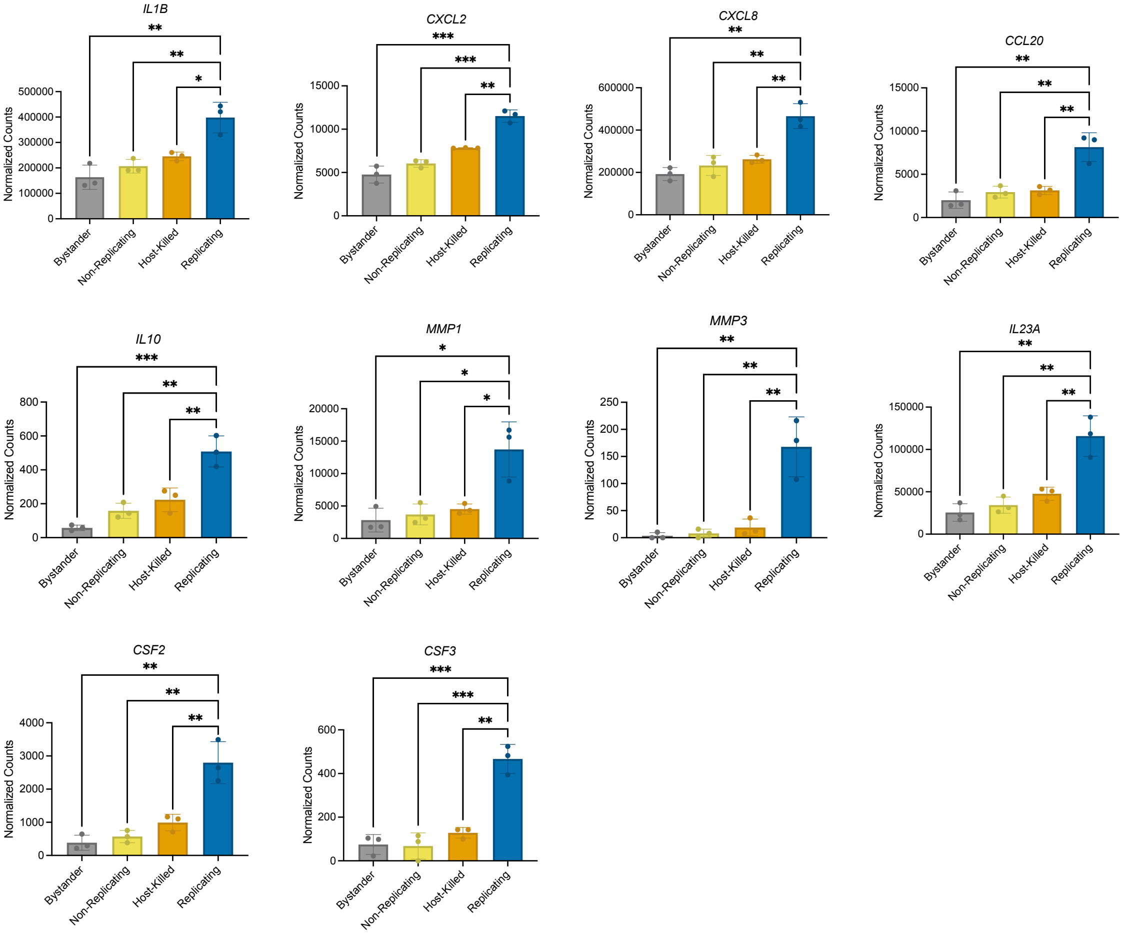

Figure S5: The ten hub genes from the turquoise (replicating) module are significantly more highly expressed in the population with replicating *S*. Typhi than in the other challenged populations.

Gene expression data shown in terms of transcript counts normalized by DESeq2 are shown for *IL10*, *IL23A*, *CSF3*, *CSF2*, *CXCL8*, *CXCL2*, *IL1B*, *CCL20*, *MMP1*, *MMP3*, which are the ten hub genes from STRING protein-protein network analysis based on the Maximal Clique Centrality calculation. One-way repeated measures ANOVA was used to assess significant differences in gene expression relative to the replicating population (* indicates $p < 0.05$; ** indicates $p < 0.01$; *** indicates $p < 0.001$).

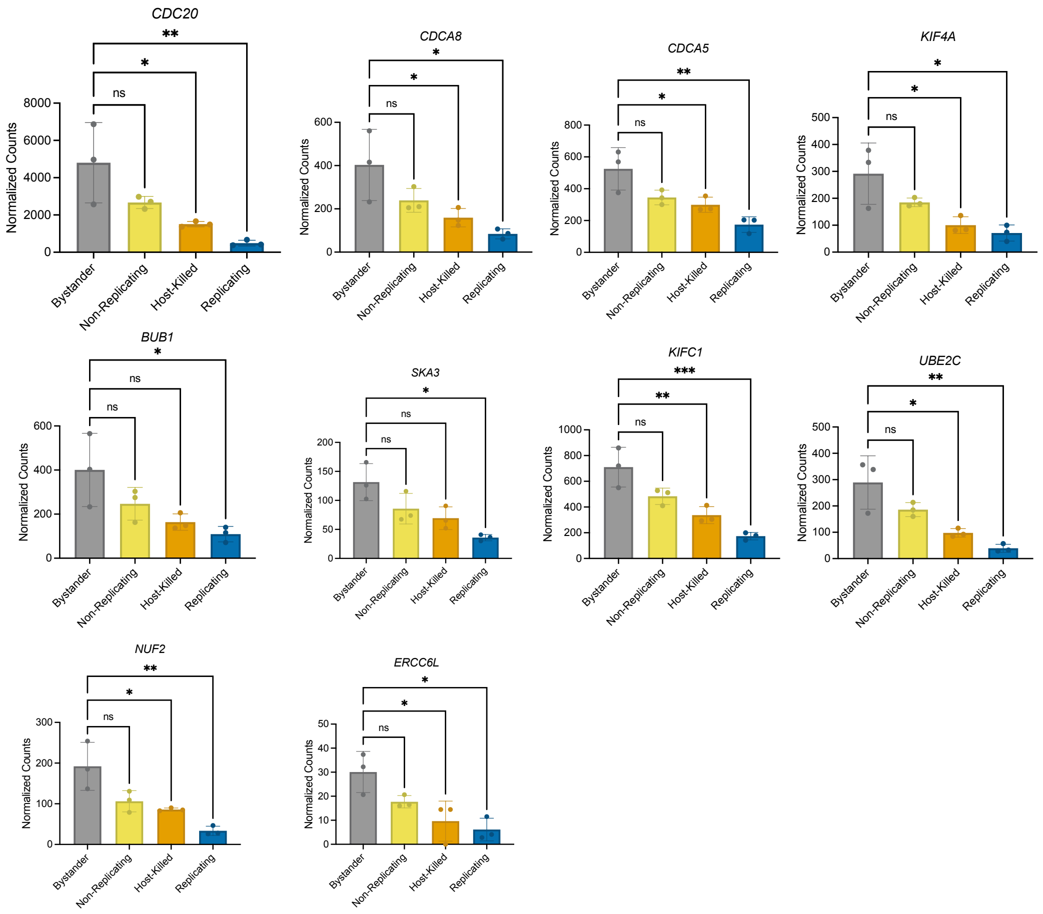

Figure S6: The ten hub genes from the blue (bystander) module are not expressed to a significantly higher degree in the bystander population compared to the population with non-replicating *S*. Typhi.

Gene expression data shown in terms of transcript counts normalized by DESeq2 are shown for *CDC20*, *CDCA8*, *CDCA5*, *ERCC6L*, *KIF4A*, *BUB1*, *SKA3*, *KIFC1*, *UBE2C*, *NUF2*, and *ERCC6L*, which are the ten hub genes from STRING protein-protein network analysis based on the Maximal Clique Centrality calculation. One-way repeated measures ANOVA was used to assess significant differences in gene expression relative to the bystander population (* indicates $p < 0.05$; ** indicates $p < 0.01$; *** indicates $p < 0.001$).

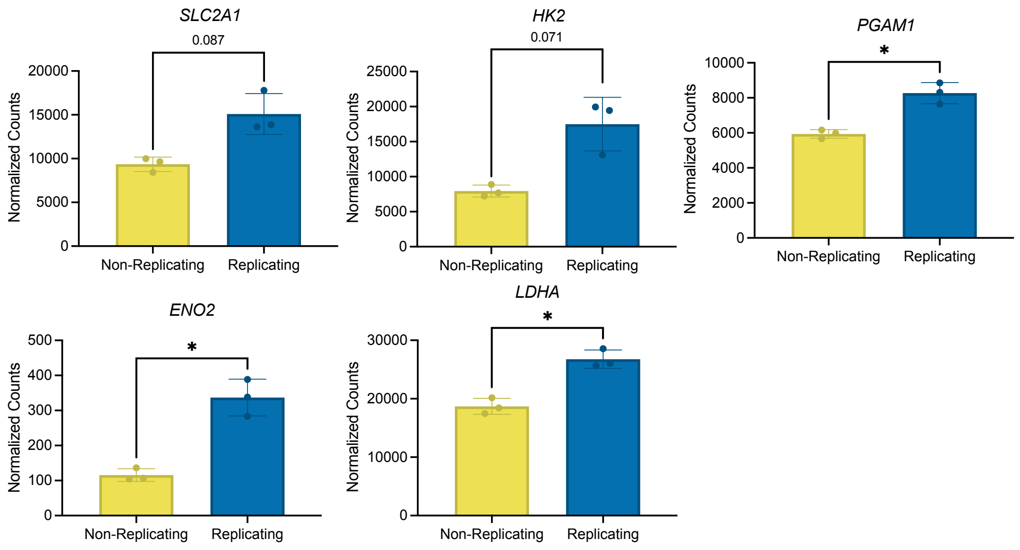

Figure S7: Macrophages harboring replicating *S*. Typhi are characterized by higher expression of glucose metabolism genes relative to macrophages harboring non-replicating *S*. Typhi.

Gene expression data in terms of transcript counts normalized by DESeq2 are shown for *SLC2A1*, *HK2*, *PGAM1*, *ENO2*, and *LDHA*. Paired *t*-test was used to assess significant differences in gene expression (* indicates $p < 0.05$; ** indicates $p < 0.01$; *** indicates $p < 0.001$).
